# Usnic Acid Potassium Salt: Evaluation of the Acute Toxicity and Antinociceptive Effect in Murine Model

**DOI:** 10.3390/molecules24112042

**Published:** 2019-05-28

**Authors:** Hallysson Douglas A. Araújo, José G. Silva Júnior, João R. Saturnino Oliveira, Maria Helena M. L. Ribeiro, Mônica C. Barroso Martins, Marcos A. Cavalcanti Bezerra, André Lima Aires, Mônica C. P. Azevedo Albuquerque, Mário R. Melo-Júnior, Nicodemos T. Pontes Filho, Eugênia C. Pereira, Diego J. Raposo Silva, Janaína V. dos Anjos, Emerson Peter S. Falcão, Nicácio H. Silva, Vera L. Menezes Lima

**Affiliations:** 1 Centro de Biociências–Departamento de Bioquímica, Universidade Federal de Pernambuco, Avenida Prof. Moraes Rego, 1235, Cidade Universitária, Recife, PE 50.670-501, Brazil; juniorguedes18@hotmail.com (J.G.S.J.); ricardhis@gmail.com (J.R.S.O.); monicabarmartins@hotmail.com (M.C.B.M.); nhsilva@uol.com.br (N.H.S.); 2 Laboratório de Imunopatologia Keizo Asami (LIKA), Universidade Federal de Pernambuco. Avenida Prof. Moraes Rego, 1235 Cidade Universitária, Recife, PE 50.670-501, Brazil; maria.mlima@ufpe.br (M.H.M.L.R.); andrelima26@gmail.com (A.L.A.); jcmonica@globo.com (M.C.P.A.A.); mariormj@gmail.com (M.R.M.-J.); ntpf@ig.com.br (N.T.P.F.); 3 Centro de Biociências–Departamento de Biofísica e Radiobiologia, Universidade Federal de Pernambuco. Avenida Prof. Moraes Rego, 1235 Cidade Universitária, Recife, PE 50.670-501, Brazil; macbezerra.ufpe@gmail.com; 4 Centro de Ciências da Saúde–Departamento de Medicina Tropical, Universidade Federal de Pernambuco. Avenida Prof. Moraes Rego, 1235 Cidade Universitária, Recife, PE 50.670-501, Brazil; 5 Centro de Ciências da Saúde–Departamento de Patologia, Universidade Federal de Pernambuco. Avenida Prof. Moraes Rego, 1235 Cidade Universitária, Recife, PE 50.670-501, Brazil; 6 Departamento de Ciências Geográficas, Centro de Filosofia e Ciências Humanas, Universidade Federal de Pernambuco, Recife, PE 50.740-530, Brazil; 7 Departamento de Química Fundamental, Universidade Federal de Pernambuco, Recife, PE 50.740-540, Brazil; sherlockmang@yahoo.com.br (D.J.R.S.); janaina.anjos@ufpe.br (J.V.d.A.); 8 Laboratório de Síntese e Isolamento Molecular, Centro Acadêmico de Vitória de Santo Antão, Universidade Federal de Pernambuco, Vitória de Santo Antão, PE 55.608-680, Brazil; emerson_falco@yahoo.com.br

**Keywords:** lichen, *Cladonia substellata*, usnic acid derivatives, antinociceptive activity (phase I and II), toxicological survey, soluble drug, histopathology

## Abstract

To obtain usnic acid potassium salt (PS-UA), the usnic acid (UA) was extracted and purified from the lichen *Cladonia substellata*, and modified to produce PS-UA. The structure was determined by ^1^H-NMR, IR and elemental analysis, ratified through computational models, as well as identification the site of K^+^ insertion in the molecule. Antinociceptive activity was detected through contortions in mice induced by acetic acid and formalin (phases I and II) after treatments with 10 and 20 mg/kg of PS-UA, indicating interference in both non-inflammatory and inflammatory pain. After oral administration at doses of 500, 1000 and 2000 mg/kg, no deaths of mice with treatments below 2000 mg/kg were observed. Except for body weight gain, food and water consumption decreased with treatments of 1000 and 2000 mg/kg, and the number of segmented leukocytes was higher for both treatments. Regarding serum levels, cholesterol and triglycerides decreased, however, there was an increase in hepatic transaminases with both treatments. Liver and kidney histological changes were detected in treatments of 2000 mg/kg, while the spleen was preserved. The PS-UA demonstrated antinociceptive activity while the acute toxicity at the concentration of 2000 mg/kg was the only dose that presented morphological changes in the liver and kidney.

## 1. Introduction

The lichen biota constitutes about 8% of total vegetation and is used in different cultures around the world [1]. Lichens present in their symbiotic structure at least one fungus (heterotrophic mycobiont) and one or more algae or cyanobacteria (autotrophic photobiont), which produce secondary metabolites. About 1000 lichen metabolites have been described, of which more than 80% are exclusive to the mononuclear, aromatic, depsidone, diphenyl ether, and dibenzofuran classes [2].

Ethnopharmacological research has listed the use of lichens and their derivatives in folk medicine for a wide range of pharmacological activities, demonstrating astringent, laxative, anticonvulsant, antiemetic, antiasthmatic, anti-inflammatory, and antibiotic properties, as well as uses against diarrheal diseases, skin diseases, epilepsy, convulsions, sore throats, and toothaches and also for the treatment of cardiovascular, respiratory, and gastric diseases [2,3]. The great interest in finding remedies from natural resources is directly associated with the high cost of synthetic drugs, which mainly affect economically disadvantaged populations seeking primary care in poor or developing countries [4]. 

Among the secondary metabolites of lichens, there is usnic acid (UA) (2,6-diacetyl-7,9-dihydroxy-8,9b-dimethyl-1,3(2H,9bH)-dibenzo-furandione C_18_H_16_O_7_), which has a yellow color and is metabolized by several species widely distributed in tropical and subtropical countries [5]. UA is naturally found in two enantiomeric forms, differentiated by the orientation of the methyl group located at position 9b. UA is also attributed with a variety of pharmacological potential, having gastroprotective, immunostimulatory, antiviral, antimicrobial, anti-inflammatory, antiprotozoal and antitumor activities [1,6,7].

In addition, pharmacological studies have been carried out to test and develop natural compounds isolated from lichens for human use related to pain, which is a neurological sign with nociceptive perception due to unpleasant sensations stimulated by subjective and objective factors of the system. Both factors are directly related to the mechanisms of pain response in stimulation to peripheral nerve responses (pain in the first minutes) or more pronounced stimulation (pain lasting ≥ 10 min after injury) [8]. In this regard, Okuyama et al. [9] evaluated diffractaic acid and UA in a murine pain model and observed that UA had a very considerable effect when using the acetic acid contortion method. However, UA has the disadvantage of presenting low aqueous solubility and high toxicity due to its hydrophobic characteristics related to its physico-chemical properties [10]. As a result, several studies have aimed at improving the properties of UA, such as the inclusion of usnic acid β-cyclodextrin [11], usnic acid-polyacrylamide [12], solid lipid nanoparticles [13], PLGA microsphere encapsulation [14], and the incorporation in polyurethanes [15,16] and in liposomes [17].

Therefore, it is important to introduce new, effective methods to increase the solubility and dissolution rate of drug candidates in order to improve their oral bioavailability, to increase the predictability of responses, and/or to reduce the dose [18,19,20]. Thus, one of the alternatives for increasing the bioavailability of UA is to chemically modify it in the form of usnic acid potassium salt (PS-UA), which optimizes not only its solubility but also its toxicity without decreasing its biological potential [21,22,23,24,25]. Although lichen substances are widely mentioned in folk medicine, studies of their chemically modified bioactive molecules are incipient. UA in the form of PS-UA, inhibits invasion and metastasis in colorectal cancer [21]; its efficiency against adult snails [22] and different embryonic stages of *Biomphalaria glabrata* (vector of schistosomiasis) [23,24] has been demonstrated, as well as good in vitro activity against adult couples worms of *Schistosoma mansoni* at concentrations of 100 and 50 μM in a 24-h interval. With respect to cytotoxicity, PS-UA was non-toxic to peripheral blood mononuclear cells (PBMC) at the same concentrations [25].

However, effects on pain, as well as its acute toxicity, have not yet been reported. In this sense, the objective of this study was to evaluate, for the first time, the antinociceptive activity of PS-UA and its action mechanism through the nociceptive pathway and to determine its acute toxicity in a murine model by analyzing the behavioral, hematological, biochemical and histological parameters.

## 2. Results and Discussion

### 2.1. Chemical Analysis of UA and PS-UA

UA TLC analysis showed a Rf value of 0.84, while in the HPLC the retention time (RT) was 20.46 minutes, consistent with the standard UA RT. The chemical structures of UA and PS-UA were confirmed by ^1^H-NMR, ^13^C-NMR (400 MHz, Acetone-d6) and IR (KBr) and ^1^H-NMR, IR and elemental analysis respectively (see supplementary data). The optical rotation of UA was $\alpha_{25}^{D}$ +478.2200 (c 1.0 acetone). In this way, the UA used in our study is dextrorotary.

**Figure 1:**
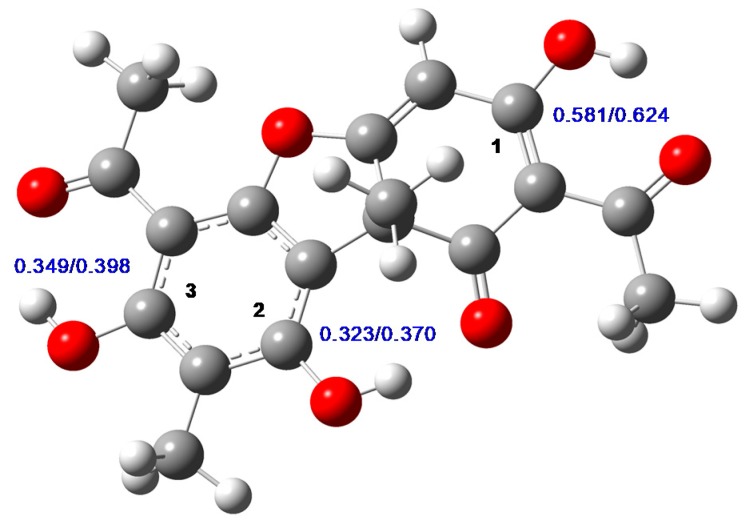
Structure of the usnic acid (most stable tautomer) from AM1 geometry optimization. The carbons bonded to hydroxyl groups, and the respective ESP (electrostatic potential) charges (B3LYP/6-31g+(d,p)) of each (ideal gas/water) are indicated.

PS-UA is characterized by the presence of K^+^ counterion, what leaded to be UA a water-soluble derivative. Huneck and Yoshimura [26] described the PS-UA as a phenolic salt, having its first deprotonation at the OH group in C1 (Figure 1). Guo et al. [1] had mapped the pKa of OH groups of UA molecule, and they also considered the most acid group should be the OH in C1. However, crystallographic evidences mentioned by Ribár et al. [27] suggest the possibility of linkage of the potassium counterion to the beta-diketone group. In both cases, the ressonance of keto groups could desplace the charge, leading the proposed structure for the salt.

In order to confirm the PS-UA structure, we performed some calculations using the Gaussian 09 program [28]. The geometry optimization of tautomer UA, which is the most stable according to previous theoretical studies [29,30], was performed using the AM1 semi-empirical method, and the electronic part of equilibrium geometries was obtained in accordance with the ab initio level of theory. The same procedure was applied to the anions of UA, formed after each possible deprotonation (Figure 1)**,** with or without a previous proton abstraction, in both ideal gas and water.

The optimized geometry of UA tautomer of usnic acid is presented in Figure 1, which is similar to previous calculations [30]. From the ESP (electrostatic potential) charges also presented in this figure, we observe that carbon 1 is the most electrophylic as compared to carbons 2 or 3, in both ideal gas and aqueous solution. This indicates a first deprotonation in the hydroxyl group bonded to this carbon, since it can more effectively stabilize the oxygen charge after anion formation. In order to confirm this preference in terms of the stability of each anion, we compared the sum of the electronic energy (from B3LYP) with the thermal correction term (from AM1) of each anion of UA, formed by deprotonation on carbons 1, 2 or 3 (leading to the anions A1, A2, and A3, respectively) according to the numbering on Figure 1. Both in ideal gas and in aqueous solution, the anion created by the deprotonation of the nonphenolic OH is the most stable. The following orders of stability, with the energies relative to such anion, were found: (a) A1 > A2 (6.76 kcal mol^−1^) > A3 (18.23 kcal mol^−1^) in ideal gas and (b) A1 > A2 (7.81 kcal mol^−1^) > A3 (10.94 kcal mol^−1^) in water. These data support the hypothesis that the most acid proton belongs to the OH group bounded with carbon 1, as previous calculations using the PCM implicit solvent model have verified [30].

**Figure 2:**
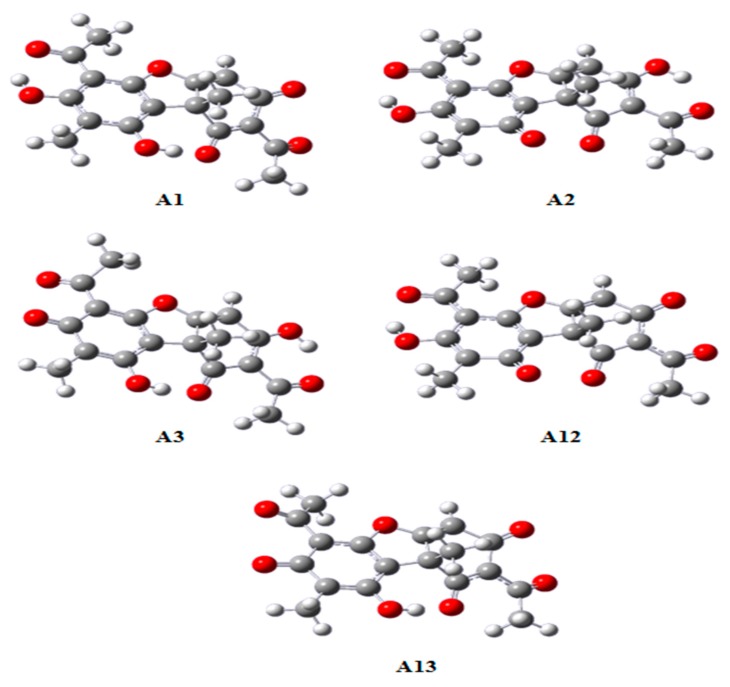
Equilibrium structures of usnic acid anions. A1, A2 and A3 formed after one single deprotonation and anions A12 and A13 formed after two deprotonations.

After the first deprotonation, the second can occur on carbon 2 (leading to anion A12) or carbon 3 (leading to anion A13). It was found that the anion A12 is more stable than A13, by a difference of 2.28 kcal mol^−1^ in ideal gas and 1.72 kcal mol^−1^ in water. Therefore, the anion formation order (as a result of sequential deprotonation) should be A1 → A12 → A13, where A13 is the anion which charge -3, the result of three successive deprotonations. The same trend was verified by Galasso [30]. This implies the probable position for K^+^ in the usnic acid salt near the negatively charged oxygen bonded to carbon 1 (Figure 2).

Natural derivatives are important sources of raw materials in obtaining molecules in the composition and development of promissory compounds, when the aim is the development of new drugs. In addition, they can also allow structural modifications (syntheses) from these substances, potentiating their biological activity, thus leading to an optimization of therapeutic activity [4,18,21,22,23,24,25,31,32]. Therefore, we contribute with the first report of PS-UA obtained from UA isolated from *Cladonia substellata* (lichen). This derivative showed low acute toxicity and high antinociceptive activity at low concentrations in a murine model. This result, in addition to being a novelty, can be considered as useful for further studies that involve tropical and neglected diseases.

### 2.2. Signs of Toxicity and Behavioral Analysis of Mice after PS-UA Treatment

The toxic effects of PS-UA through behavioral analyses in mice are shown in Table 1. 

The acute toxicity assay is usually performed to safely determine the dose ranges of substances, but it can also provide initial information on the mechanisms of toxicity or homeostatic changes [33]. The negative control group did not show behavioral changes, whereas all tested concentrations (500, 1000 and 2000 mg/kg of PS-UA) showed at least some effect, either stimulant, depressant or change in depression versus agitation. According to Nascimento et al. [34], systemic toxicity is demonstrated through alterations, emphasizing those that affect both the central and the autonomous nervous system, as well as somatomotor activity. Moderate and high piloerection and prostration effects were observed in the initial period after administration of PS-UA at 500, 1000, and 2000 mg/kg. On the other hand, the reversion of these initial clinic signals after 1 h of administration was observed.

Mice treated with 2000 mg/kg showed the highest number of behavioral changes and were the only ones with mortality at 48 and 72 h intervals, but this was only a 40% mortality rate. In this context, we can observe that the PS-UA LD_50_ (although it was not possible to calculate) was shown to be higher than that of UA 838 mg/kg as reported by Sigma-Aldrich [35]. These results corroborate with the Organization for Economic Cooperation and Development (OECD) technical specifications [36], which indicate that substances with an LD_50_ of more than 1000 mg/kg orally are considered safe or slightly toxic. Thus, we can observe that after the transformation of UA into PS-UA, the result was a drug with greater bioavailability and a much lower toxic effect.

The toxicity mechanism of UA has not yet been completely elucidated. However, Han et al. [37], Pramyothin et al. [38], and Joseph et al. [39] have already signaled that UA acts by altering the integrity of the cellular membrane (lipophlilic characteristic of the drug), allowing the liberation of hepatospecific enzymes, mainly transaminases. In addition, it causes the destruction of mitochondrial function (complex I to IV of electron transporting chain), exhibiting the loss of control of the cell respiration and synthesis of ATP. These studies indicated that UA has a similar effect to that of carbon tetrachloride, which evolves the generation of free radicals, which results in injuries in both cell and mitochondrial membranes, expression of genes associated to lipid peroxidation, Krebs cycle, and apoptosis, increasing the production of reactive oxygen through the electron transporting chain, leading to cell death.

In the USA, these aforementioned hepatocellular damages were confirmed after using LipoKinetix^®^, a dietary supplement containing UA. The users who consumed this supplement showed liver acute collapse, one needing a hepatic transplant. Another patient, after 8 weeks, had recovered due to specialized accompaniment [40]. Similarly, two other users that had consumed three capsules a day of UDP-1 (dietary supplement with 150 mg of UA, 525 mg of carnitine, and 1050 mg of calcium pyruvate per capsule) developed severe hepatoxicity after three months of use and exhibited fulminant hepatic failure. In one of the cases, liver transplant was necessary. Histopathological analysis showed cytoplasmic lymphocytic infiltrates and areas with necrosis in the liver of patients who had used UDP-1 [41].

No significant differences (*p* > 0.05) in body weight gain were observed for any treatment. In relation to food and water consumption, it was observed that only the mice in the control group and treated with 500 mg/kg did not present a significant difference. In contrast, mice treated with PS-UA at 1000 and 2000 mg/kg showed a reduction (*p* < 0.001) in food consumption and less water consumption than control mice Table 2. This may be directly related to the metabolic changes observed during treatment.

The evaluation of these parameters is of great importance, mainly for substances with therapeutic purposes such as analgesics and anti-inflammatory agents, since adequate intake of nutrients and water are essential for the potentiality of the drug and/or to avoid gastrointestinal irritations mainly caused by the administration of these drugs over a prolonged period [42]. At present, we already have in-depth studies with humans on these mechanisms where the objective was to more precisely demonstrate drug nutrient interactions and their effects on the bioavailability of the drugs [43,44].

### 2.3. Haematological and Biochemical Analyses

The hematopoietic system is one of the most sensitive targets for toxic compounds and serves as an important indicator, being reliable to evaluate health and safety conditions and very well defined for the pathophysiological changes of blood hemostasis in both humans and the murine model [33]. In this report, both the red blood cell and leukocyte profiles were analyzed as important toxicological indices. The results described in Table 3 show that PS-UA did not cause severe alterations (hyperchromic/hypochromic, microcytic/macrocytic and anemia) in the red series.

However, it stimulated the immune system, with an increase in segmented cells for treatments of 1000 and 2000 mg/kg. This increase in the number of segmented leukocytes has also been reported in other studies evaluating the acute toxicity of organic extracts [45,46]. Recently, a study by Oliveira et al. [47], evaluating the acute toxicity of *Pilosocereus gounellei* saline extract on mice, observed a significant increase in the number of segmented cells at a 2000 mg/kg treatment and total leukocytes at 5,000 mg/kg. According to Kumar et al. [48], the leukogram is the part of the hemogram that investigates quantitative and/or morphological alterations of the leukocyte series. Its quantitative abnormalities are leukocytosis (increase) and leukopenia (decrease). Leukocytosis can be attributed to physiological and/or pathological factors such as tissue damage (inflammation) in the example of the recurrent liver of the exacerbated metabolism of substances which at very high concentrations present toxicity.

The results of the biochemical analyses are described in Table 4. The treatments with PS-UA revealed decreased serum levels of cholesterol and triglycerides in the 1000 and 2000 mg/kg treatments, compared to the control group. This decrease can be directly related to the decrease of food intake and is not necessarily caused by metabolic changes (Table 2). Meanwhile, the values of the biochemical levels of ALT (alanine aminotransferase), AST (aspartate aminotransferase), alkaline phosphatase, and creatinine increased and varied significantly (*p* < 0.05) between the groups for the 1000 and 2000 mg/kg treatments (Table 4). These increased serum levels indicate liver hepatotoxicity and impaired renal function for PS-UA.

### 2.4. Histopathological Analyses of the PS-UA Treatments

The PS-UA treatments did not show statistically significant differences (*p* > 0.5) for liver, kidney, and spleen weights at doses of 500 and 1000 mg/kg compared to the control group: The control group was 1.51 ± 0.14, 0.31 ± 0.03, and 0.12 ± 0.25; PS-UA 500 mg/kg was 1.53 ± 0.19, 0.31 ± 0.01, and 0.11 ± 0.01; and PS-UA 1000 mg/kg was 1.83 ± 0.22, 0.33 ± 0.46, and 0.11 ± 0.03. However, in the treatment with 2000 mg/kg PS-UA, there were changes in the liver and kidney morphologies (increase in size/weight) compared to the control group (3.06 ± 0.42 (*p* < 0.001) and 0.44 ± 0.03 (*p* < 0.001)). Only the spleen weight (0.12 ± 0.02) remained similar to the control.

**Figure 3:**
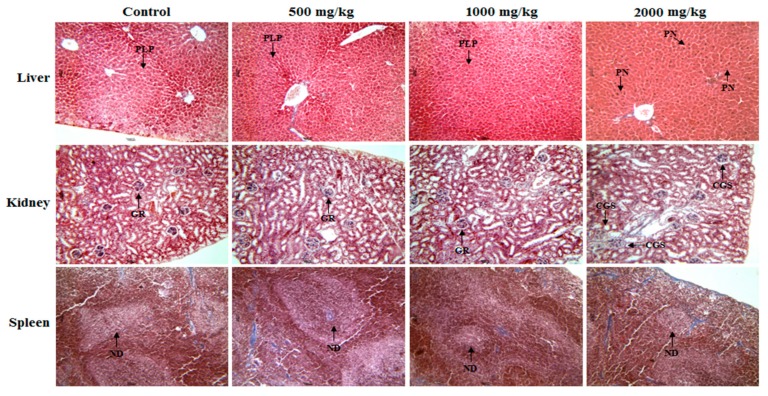
Representative photomicrographs of the livers, kidneys, and spleens of control mice (oral saline) and those treated with PS-UA (500, 1000, and 2000 mg/kg; oral administration). The liver with preserved liver parenchyma (PLP) and without significant histopathological changes compared with in the treatment of 2000 mg/kg, the liver presented pyknotic nucleus (PN). Kidneys: Renal glomeruli (GR) and contorted tubules without changes are visible in the control and groups treated with PS-UA at the 500 and 1000 mg/kg doses, while in the 2000 mg/kg treatment, the kidneys showed changes in the glomerular space, with morphological distortions. Spleen: The lymphatic nodes (ND) are well-defined in the control and treated groups. Hematoxylin and eosin staining was used. All images are with 100× magnification.

The histopathological analysis of liver, kidney, and spleen for the control group and the 500, 1000, and 2000 mg/kg treatments with PS-UA can be seen in Figure 3. 

The normal architecture of hepatic, renal and splenic organs are observed in the control and in treatments with 500 and 1000 mg/kg of PS-UA. In the liver, the liver parenchyma was preserved and there were no significant histopathological changes. In the kidney, a glomerular architecture could be observed and Bowman’s capsule and renal tubules were without inflammatory changes. In the spleen, splenic structure was preserved with well-defined lymphoid follicles in both treated and control groups. On the other hand, at the 2000 mg/kg dose histological alterations in the hepatic and renal tissues were observed. In the liver, the destructive fragmentation of the hepatocyte nucleus, a phenomenon called pynotic nucleus (PN) and characterized by the irregular distribution of chromatin from programmed cell death (apoptosis), was indicative of future tissue necrosis [48]. In the kidney there were changes in the glomerular space, with morphological distortions. As the kidney expands, it restricts and distorts the glomerular capillaries, decreasing the filtration surface of the capillary. These changes demonstrate why the mice treated with 2000 mg/kg consumed 50% less water than the control group. In this situation, the lesions may obliterate the glomerulus, with consequent reduction in renal function due to the lack of water intake. This was reported by a significant increase in creatinine value indicating renal change (Table 4). When these changes persist, it can lead to chronic or even terminal renal failure [48]. However, the splenic tissue did not present significant changes with defined and preserved lymph nodes.

Acute toxicity assay is usually conducted to determine the safety of a substance at determined dose ranges, but it can also provide initial information on toxicity mechanisms through the investigated parameters, mainly for the development of drugs with a pharmacological profile, whose therapeutic finality encompasses several diseases, mainly those ones that include pain, but showing less adverse effects and being effective at low concentrations.

### 2.5. Antinociceptive Activity

**Figure 4:**
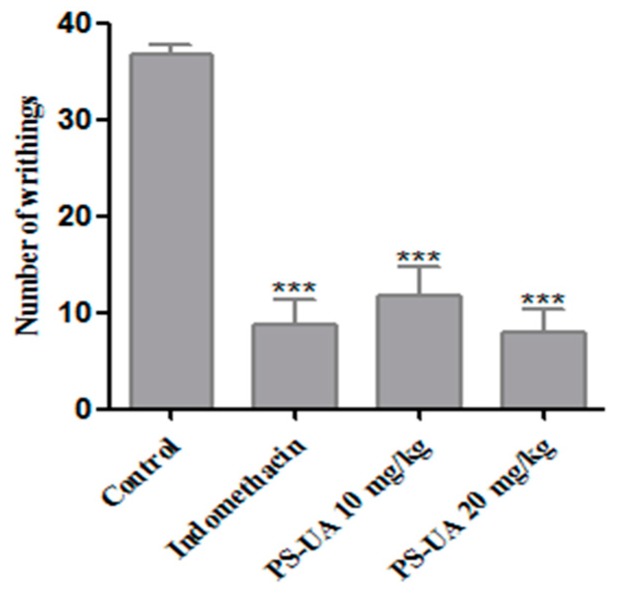
The antinociceptive effect of PS-UA (10 and 20 mg/kg; oral administration) and the reference drug indomethacin (20 mg/kg; intraperitoneal administration) in the acetic acid-induced writhing assay. The bars represent the mean numbers of writhing ± SD. (***) indicates a significant difference (*p* < 0.001) in the number of contortions versus the control.

The antinociceptive activity of PS-UA was evaluated by two methods: acetic acid-induced contortion and the formalin test. The drug chosen was the reference indomethacin, where it showed an antinociceptive effect as expected. However, the concentrations with antinociceptive action (10 and 20 mg/kg of PS-UA) were associated to the doses that exhibited a macroscopic reduction of hepatic tissue, and hepatoprotector effect, after inhibition of hepatic metastasis of colorectal cancer in the murine model [21]. The treatments performed with doses of 10 and 20 mg/kg of PS-UA significantly inhibited (*p* < 0.001) the number of abdominal contortions induced by the intraperitoneal administration of acetic acid, when compared to the control group (Figure 4).

The groups treated with either 10 and 20 mg/kg of PS-UA dose presented a reduction of 68% and 78%, respectively, but with no significant difference (*p* > 0.5) between the concentrations. The contortions induced by acetic acid are caused by the irritation of the acid injected intraperitoneally involve the stimulation of peripheral nociceptors and consequently causes behavioral reactions triggering the release of mediators such as P substances, bradykinins, prostaglandins and proinflammatory cytokines. These, in turn, stimulate peripheral nociceptors and neurons sensitive to inflammatory mediators [49]. Prostaglandin and bradykinin, for example, cause changes not only in specific receptors (TRPV1) coupled to the ion- and binder-dependent channels via cAMP activation but also in the protein kinases A (PKA) and C (PKC), reducing neural membrane post-hyperpolarization time and causing a reduction in the threshold for firing of the nerve fiber [50]. Regarding the antinociceptive activity of UA, Okuyama et al. [9] reported analgesic effects of acetic acid-induced contortion and tail pressure in mice for treatments of 30 and 100 mg/kg, where they concluded that acetic acid-induced pain was only decreased at a concentration of 100 mg/kg, while for tail pressure both treatments presented significant analgesic results. Therefore, it is believed that the potentiality of the analgesic activity of PS-UA by the acetic acid-induced contortion method in treatments of 10 and 20 mg/kg can be attributed to the K^+^ radical, since the K^+^ present in the structure of PS-UA is the only element that differentiates it from UA and confers hydrophilic characteristics to PS-UA, increasing its bioavailability and its pharmacological effects on pain.

**Figure 5:**
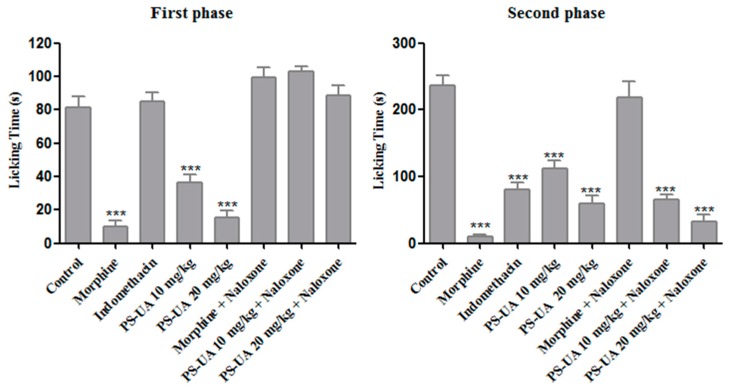
The antinociceptive effect of PS-UA (10 and 20 mg/kg; oral administration) and the reference drugs, indomethacin (20 mg/kg; intraperitoneal) and morphine (10 mg/kg; oral administration), in both phases of the formalin assay. The bars represent the mean time spent by the mice licking their paws ± SD. The involvement of opioid receptors in the antinociceptive effect was also evaluated by administering naloxone (5 mg/kg, intraperitoneal) to the mice 30 min before the administration of PS-UA or morphine. (***) indicates significant difference (*p* < 0.001) in the licking time versus the control.

In the formalin test, the antinociceptive activity of PS-UA was also detected in the 10 and 20 mg/kg treatments, acting very significantly (*p* < 0.001) in the neurogenic and inflammatory phases (Figure 5), and reducing the time mice spent licking their paw by 55% and 81% in the first phase (neurogenic pain) in the respective treatments. In the second phase (inflammatory pain), PS-UA showed an antinociceptive effect again at both doses, reducing the time of paw licking by 53% and 73%, respectively. On the other hand, indomethacin suppressed the response only in the second phase by 66%, while morphine was active in both phases corresponding to an 88% and 95% reduction, respectively. When PS-UA was associated with naloxone it suppressed the response of inflammatory pain only in the second phase for both treatments, corresponding to 72% and 86% respectively. Naloxone is a non-selective opioid receptor antagonist that competes directly with morphine for catalytic sites/linkers. This suggests that the antinociceptive effect of PS-UA is mediated by the activation of opioid receptors, demonstrating a strong antinociceptive effect in neurogenic (non-inflammatory) pain and inflammatory pain [8,51]. 

Opioid and non-opioid analgesics are the mainstay of pain management. Antidepressants, anticonvulsants and other drugs active on the central nervous system can be used for chronic, neuropathic pain or even in the stages of terminal illnesses, being the first-line treatments for some conditions with the purpose of reducing pain and related incapacities [52]. The nociceptive drugs that act at the level of the central nervous system in the first phase are the main agents that act in the neurogenic phase of nociception and present an action mechanism characterized by the direct activation of the sensory C fibers through the cationic potential channel of the transient receptor, subfamily A, member 1 (TRPA1) [53,54,55].

In inflammatory pain (second phase), the nociceptors are sensitized by the action of chemical substances, called algiogenic agents, present in the tissue environment. This results in the release of the inflammatory mediators acetylcholine, bradykinin, histamine, serotonin, substance P, leukotriene, platelet activation factor, acid radicals, potassium ions, prostaglandins, thromboxane, interleukins, tumor necrosis factor (TNFα), nerve growth factor (NGF) and cyclic adenosine monophosphate (cAMP) [50]. An important finding is the comparison of the antinociceptive effect of morphine with the treatment at the 20 mg/kg dose of PS-UA in the first and second phases, where no significant difference (*p* > 0.05) was found between the two drugs. Thus, we can affirm from the results that PS-UA was effective in reducing local (first stage, neurogenic pain) and inflammatory (second phase) pain, interfering in the central nociceptive pathway, which corroborates its dual efficacy in both the acetic acid-induced abdominal contortions and the formalin test.

## 3. Material and Methods

### 3.1. Lichen Collection

The species *Cladonia substellata* Vanio (1887) was collected in February 2015 in the city of Mamanguape, Paraíba (PB-Brazil) 6°42′1.5″ S 35°8′3.3″ W. A voucher specimen was deposited in the herbarium Geraldo Mariz, Department of Botany of the Federal University of Pernambuco (UFPE), Recife/PE, Brazil (voucher n° 77.474). 

### 3.2. Extraction and Isolation of Usnic Acid and its Modification into Potassium Salt

*C. substellata* (120 g) was extracted with diethyl ether (5×) in a Soxhlet apparatus at 40 °C for 16 h. Each extraction was dried at room temperature (28 ± 3 °C). Subsequently, the extracts were filtered and concentrated to dryness on a rota-evaporator coupled to a water bath at 37 °C. UA was isolated and purified on a silica gel column (70–230 mesh) and eluted with chloroform-hexane (80:20 *v*/*v*). The fractions obtained were monitored by thin-layer chromatography (TLC), TLC: 0.1 mg samples of the fractions obtained were dissolved in acetone (0.5 mL). Then, 1 μL of the solution was applied to a silica gel plate (Gel 60 F254+366 Merck^®^, Darmstadt, Germany) measuring 20 × 20 cm. TLC assays were carried out under increasing polarity conditions using solvent system A (toluene/dioxane/acetic acid, 36:9:1, *v*/*v*/*v*) for the UA. The spots were observed under ultraviolet light (256–366 nm) and visualized on the plate (Fisatom model 509, São Paulo, Brazil), after spraying of 10% sulfuric acid, and heated at 50 °C for 20 min. The composition was evaluated by the determination of the retention factor (Rf) and comparison with the standard single acid [23]. High-performance liquid chromatography (HPLC), HPLC: A Hitachi chromatograph (655 A-11, Tokyo, Japan) was coupled to a UV detector (655 A-11, Tokyo, Japan) at 254 nm and a reverse phase column (RP 18 MicroPack MCH-18, 300-4 mm, Berlin, Germany). The UA was dissolved in diethyl ether (Merk^®^ KGaA, Darmstadt, Germany) at a concentration of 1.0 mg mL^−1^ and injected. The mobile phase consisted of methanol/deionized water/acetic acid (80:19.5:0.5 *v*/*v*/*v*) with a flow of 1.0 mL min^−1^, 0.04 attenuation at room temperature (28 ± 3 °C). The substance was identified based on its retention time (RT) and peak area when compared to standard UA [23]. The molecular structure was determined by proton nuclear magnetic resonance (^1^H-NMR) and carbon (^13^C-NMR) obtained at 400 MHz in acetone d-6 (Varian UNITY spectrometer, Santa Clara, CA, USA), while infrared spectroscopy (IR) analyses were performed in a Bruker Fourier spectrometer (model IFS 66, Ettlingen, Germany) with KBr disks [23,25]. In addition, the optical rotation of UA was determined in a Jasco P2000 polarimeter (Jasco Incorporated, Easton, MD, USA) at the Analytical Centre of Fundamental Chemistry Department of Federal University of Pernambuco.

After chemical characterization and confirmation of the purity of UA, 1 g of the compound was added to 800 mL of water milli-q, and 5 mL KOH (5%) solution was gently dropped until complete solubilization of UA, until pH 11 was reached. The solution was frozen at −80 °C, lyophilized (reaction that give 100% yield), and stored in desiccator. The confirmation of the PS-UA structure was done through ^1^H-NMR, and IR and elemental analysis (C, H) was performed using a Perkin Elmer CHN-2400 (Waltham, MA, USA) at University of São Paulo–USP [23,25].

### 3.3. Computational Methods

All the calculations where performed using the Gaussian 09 program (Gaussian Inc., Wallingford, CT, USA) [28]. The geometry optimization of tautomer UA, which is the most stable according to previous theoretical studies [29,30], was performed with semi-empirical method AM1, and the electronic part of equilibrium geometries was obtained in accordance with the ab initio (DFT—density functional theory) level of theory, with the combination method/basis set being B3LYP/6-31g+(d,p). The same procedure was applied to the anions of UA, formed after each possible deprotonation on carbons 1, 2 or 3 (A1, A2, and A3, respectively), with or without a previous proton abstraction. The energies in the aqueous solution were obtained in accordance with the SMD (solvent model density) implicit solvent model, from the ideal gas equilibrium geometries [56]. Calculation of ESP charges of UA atoms was performed in accordance with the Breneman and Wiberg algorithm implemented by the input line (POP=CHELPG) [57], in both ideal gas and water.

### 3.4. Acute Toxicity Evaluation

Acute toxicity experiments were carried out using Swiss webster mice (32 ± 2 g) reared at the Keizo Assami Immunopathology Laboratory (LIKA) of UFPE and kept under a controlled environment (20 ± 2 °C, 12 h light/dark cycle) (libitum/Purina, São Paulo (SP)). All experimental procedures were only carried out after approval by the Animal Experimentation Ethics Committee (CEEA) of the Bioscience Center, Federal University of Pernambuco (UFPE) (Process N^o^. 23076.015163/2017-65).

The acute toxicity effect of PS-UA was performed according to the guidelines of the Organization for Economic Cooperation and Development [36]. Acute toxicity (mortality and behavioral changes) was evaluated by oral administration. PS-UA was dissolved in distilled water to avoid the effect of salting-out. Mice were divided into 4 groups (*n* = 5): a control group which received filtered water and three groups that were treated with PS-UA at doses of 500, 1000 and 2000 mg/kg. Mice were observed for 5 days. On the first day, behavioral changes were observed every 10 min for 4 h, followed by two observations one day after administration in order to record toxicity-related behaviorial signs through the parameters: respiratory frequency, piloerection, stereotyped movement, fine tremors, lifting upper train, spasms, prostation, lowering hind quarters, photophobia, fecal excretions, abdominal distension, and death [58]. From the time of treatment, variations in body weight were determined, as well as the daily consumption of water and food. On the 5th day after the start of treatment, peripheral blood was collected, then the mice were euthanized, and the liver, kidney and spleen were removed, weighed macroscopically and processed for histological evaluation.

### 3.5. Haematological and Biochemical Analyses

The blood collected was used to evaluate hematological and biochemical alterations. The following hematologic parameters were analyzed using an automatic analyzer (CELL-DYN Ruby, Lake Bluff, IL, USA) and optical microscopy (Olympus BX 41, Olympus Corporation of the Americas, Center Valley, PA, USA.): erythrocytes, hemoglobin, hematocrit, mean corpuscular volume (MCV), mean corpuscular hemoglobin (MCH), mean corpuscular hemoglobin concentration (MCHC), and total and differentiated analyses of leukocytes. For the biochemical analysis, blood was evaluated for albumin, alanine aminotransferase (ALT), aspartate aminotransferase (AST), alkaline phosphatase, total cholesterol, triglycerides, urea, and creatinine using specific kits (Labtest Diagnóstica, Lagoa Santa, Brazil).

### 3.6. Histopathological Analysis

Histological analyses of the liver, kidney, and spleen of the control and PS-UA-treated animals were performed by optical microscopy. Fragments of the organs were fixed in buffered formalin (10% *v*/*v*), then dehydrated through a graduated series of ethanol (70, 80, 90, and 100%), diaphonized with xylol, and embedded in paraffin. Histological sections (5 μm thick) were stained with hematoxylin-eosin and fitted under coverslips with Entellan resin (Merck, Darmstadt, Germany). The slides were observed under a Labomed Lx400 microscope coupled to a Moticam 1000 digital camera of 1.3 MP USB 2.0 using Motic Images Plus 2.0 software (Motic Incorporation Ltd., Hong Kong, China).

### 3.7. Evaluation of Antinociceptive Activity

#### 3.7.1. Acetic Acid-Induced Writhing Test

Female mice were separated into four groups (*n* = 6): Group I treated with oral saline (control), Group II intraperitoneal indomethacin (20 mg/kg), and Groups III and IV with PS-UA at concentrations of 10 and 20 mg/kg, respectively. Saline or PS-UA were administered via an esophageal catheter 1 h prior to acetic acid administration, while indomethacin was given 30 min before. Each animal received an intraperitoneal injection of 0.85% (*v*/*v*) acetic acid in saline and was then placed in a polyethylene box to record the latency period (time until the first writhing) and the number of writhes in the interval corresponding to 5–15 min after the injection of acetic acid (CEEA process N^o^. 23076.015163/2017-65) [59].

#### 3.7.2. Formalin Test

The procedure used was similar to that described by Ping et al. [60]. Female mice were divided into 8 groups (*n* = 6) and the following pretreatments were administered: Group I Vehicle (100 µL of water, v.o.); Group II Morphine (10 mg/kg); Group III Indomethacin (20 mg/kg); and Groups IV and V PS-UA 10 and 20 mg/kg, respectively (v.o.). All animals were treated through gavage. After 60 min, 20 μL of 2.5% (*v*/*v*) formalin in saline was injected into the subplantar region of the right hind paw of each animal. The time spent by the mouse licking its paw was recorded during the first 5 min after formalin injection (first phase neurogenic pain), as well as 15–30 min after the injection (second phase inflammatory pain). In order to evaluate the involvement of opioid receptors and the PS-UA action mechanism, naloxone (5 mg/kg, intraperitoneal) was administered 30 min earlier, morphine 10 mg/kg + naloxone for Group VI, and 10 and 20 mg/kg + naloxone for Groups VII and VIII PS-UA, respectively [61]. 

### 3.8. Statistical Analysis

The results are expressed as the means of replicates ± standard deviation (SD). Analysis of variance (ANOVA) was performed followed by Tukey’s test for multiple comparisons. A *p* value < 0.05 was adopted as the significance level.

## 4. Conclusion

PS-UA presented moderate oral toxicity to mice, since no animal death at a dose of 1000 mg/kg was detected. However, PS-UA promoted hematological, biochemical, and histopathological changes and death at the concentration of 2000 mg/kg. The 500 mg/kg treatment of PS-UA has been shown to be an interesting dose for future pharmacological investigations without toxic effects. The results also show that PS-UA had antinociceptive activity and was active against both noninflammatory and inflammatory pain at the concentrations 10 and 20 mg/kg, being associated with interference of the opioid receptor pathway. This work contributes to the knowledge of the acute toxicity of PS-UA and to its pharmacological action for pain.

## Figures and Tables

**Table 1:** The parameters related to toxic signs and the behavioral analysis in swiss female mice assessed after the oral administration of the PS-UA in doses of 500, 1000, and 2000 mg/kg.

Parameters		Treatments PS-UA (mg/kg)		
	Control	500	1000	2000
Stimulants				
Increased respiratory frequency	-	+	+	+++
Piloerection	-	++	++	++
Stereotyped movement	-	-	+	++
Fine tremors	-	-	-	+
Lifting upper train	-	+	+	+
Depressant				
Prostration	-	+++	+++	+++
Lowering hind quarters	-	-	-	++
Others				
Photophobia	-	-	-	+
Spasms	-	-	-	+
Fecal excretion	-	-	+	+
Abdominal distension	-	-	-	++
Change: Depression x Shake				
Death	-	-	-	++

- = no effect; + = low effect; ++ = moderate effect; and +++ = high effect.

**Table 2:** The water and food consumption of mice from the controls and those treated with PS-UA.

Parameters		Treatments PS-UA (mg/kg)		
	Control	500	1000	2000
Water consumed (mL)	38.75 ± 1.49	34.10 ± 2.29	27.30 ± 7.09 ^a^	19.40 ± 6.11 ^a^
Food consumed (g)	33.70 ± 1.29	30.20 ± 3.56	22.85 ± 3.40 ^a^	15.95 ± 7.59 ^a^
Weight gain (g)	32.40 ± 1.57	31.66 ± 1.50	31.60 ± 2.15	31.88 ± 1.84

Significantly different from the control: ^a^ (*p* < 0.001) for the 1000 and 2000 mg/kg. Data are the means ± standard deviations.

**Table 3:** The hematological parameters of the blood of mice treated with PS-UA.

Parameters	Treatments PS-UA (mg/Kg)			
	Control	500	1000	2000
Erythrocytes (10^6^/mm^3^)	8.81 ± 0.81	8.88 ± 0.26	8.90 ± 0.41	9.16 ± 0.72
Hematocrit (%)	45.75 ± 3.97	45.55 ± 1.12	45.65 ± 2.65	47.05 ± 3.74
Hemoglobin (g/dL)	15.81 ± 1.40	15.53 ± 0.36	15.37 ± 0.71	15.76 ± 1.01
MCV (fL)	51.90 ± 2.23	51.25 ± 1.46	52.10 ± 0.82	51.28 ± 1.89
MCH (pg)	17.90 ± 0.38	17.35 ± 0.54	17.23 ± 0.17	17.15 ± 0.41
MCHC (%)	34.25 ± 0.95	33.25 ± 0.50	33.25 ± 0.50	33.00 ± 0.81
Leukocytes (10^3^/mm^3^)	2.21 ± 0.49	2.99 ± 0.52	3.65 ± 0.63 ^a^	4.52 ± 0.96 ^b^
Segmented (%)	40.75 ± 9.35	66.50 ± 21.75	75.75 ± 12.61^a^	74.25 ± 8.53 ^a^
Lymphocytes (%)	21.03 ± 8.41	26.70 ± 4.67	28.65 ± 8.93	20.25 ± 2.14
Monocytes (%)	14.33 ± 4.93	12.33 ± 4.04	16.67 ± 2.88	14.83 ± 2.46

Significantly different from the control: ^a^ (*p* < 0.05) and ^b^ (*p* < 0.001) from the 1000 and 2000 mg/kg treatments. Data are the means ± standard deviations. MCV: mean corpuscular volume; MCH: mean corpuscular hemoglobin; and MCHC: mean corpuscular hemoglobin concentration.

**Table 4:** The biochemical parameters of the blood of mice treated with PS-UA.

Parameter	Treatments PS-UA (mg/kg)			
	Control	500	1000	2000
Albumin (g/dL)	2.02 ± 0.28	1.90 ± 0.19	1.75 ± 0.09	1.80 ± 0.18
ALT (U/L)	39.46 ± 2.94	63.39 ± 20.7	86.78 ± 7.74 ^b^	207.6 ± 25.00 ^c^
AST (U/L)	115.8 ± 6.03	143.3 ± 22.5	229.44 ± 45.1 ^a^	306.4 ± 19.4 ^c^
Total protein (g/dL)	5.22 ± 0.05	5.27 ± 0.13	4.85 ± 0.24 ^b^	4.87 ± 0.12 ^b^
Alkaline phosphatase (IU/L)	15.04 ± 2.65	19.80 ± 4.55	31.00 ± 9.39 ^b^	33.65 ± 6.74 ^c^
Urea (mg/dL)	45.62 ± 2.54	46.56 ± 2.17	47.65 ± 1.27	46.43 ± 2.25
Creatinine (mg/dL)	041.20 ± 0.06	041.60 ± 0.06	053.40 ± 0.02	064.60 ± 0.12 ^c^
Total cholesterol (mg/dL)	90.40 ± 11.95	92.60 ± 15.99	62.20 ± 14.45 ^b^	60.50 ± 3.10 ^b^
Triglycerides (mg/dL)	133.4 ± 27.36	112.8 ± 11.82	54.50 ± 7.55 ^c^	51.75 ± 8.13 ^c^

Significantly different from the control: ^a^ (*p* < 0.05), ^b^ (*p* < 0.01), and ^c^ (*p* < 0.001) from the 1000 and 2000 mg/kg treatments. Data are the means ± standard deviations. ALT: alanine aminotransferase and AST: aspartate aminotransferase.

## Supplementary Materials

The Supplementary Materials are available online.

## References

[1] Guo L., Shi Q., Fang J.L., Mei N., Ali A.A., Lewis S.M., Leakey J.E., Frankos V.H. (2008). Review of usnic acid and *Usnea barbata* toxicity. J. Environ. Sci. Health. C Environ. Carcinog. Ecotoxicol. Rev..

[2] Yousuf A., Choudhary M.I.,  Atta-Ur-Rahman (2014). Lichens: Chemistry and biological activities. Stud. Nat. Prod. Chem..

[3] Shukla K., Joshi P.G., Rawat M.S.M. (2010). Lichens as a potential natural source of bioactive compounds: A review. Phytochem. Rev..

[4] Balunas M.J., Kinghorn A.D. (2005). Drug discovery from medicinal plants. Life Sci..

[5] Ahti T. (2000). Cladoniaceae.

[6] White P.A.S., Oliveira R.C.M., Oliveira A.P., Serafini M.R., Araújo A.A.S., Gelain D.P., Moreira J.C.F., Almeida J.R.G.S., Quintans J.S.S., Quintans-Junior L.J. (2014). Antioxidant activity and mechanisms of action of natural compounds isolated from Lichens: A systematic review. Molecules.

[7] Araújo A.A.S., Melo M.G.D., Rabelo T.K., Nunes P.S., Santos S.L., Serafini M.R., Santosa M.R.V., Quintans-Júnior L.J., Gelain D.P. (2015). Review of the biological properties and toxicity of usnic acid. Nat. Prod. Res..

[8] Hutchinson M.R., Shavit Y., Grace P.M., Rice K.C., Maier S.F., Watkins L.R. (2011). Exploring the neuroimmunopharmacology of opioids: An integrative review of mechanisms of central immune signaling and their implications for opioid analgesia. Pharmacol. Rev..

[9] Okuyama E., Umeyama K., Yamazaki M., Kinoshita Y., Yamamoto Y. (1995). Usnic acid and diffractaic acid as analgesic and antipyretic components of *Usnea diffracta*. Planta Med..

[10] Ingólfsdóttir K. (2002). Usnic acid. Phytochemistry.

[11] Lira M.C.B., Ferraz M.S., Silva D.G.V.C., Cortes M.E., Teixeira K.I., Caetano N.P., Sinisterra R.D., Ponchel G., Santos-Magalhães N.S. (2009). Inclusion complex of usnic acid with b-cyclodextrin: Characterization and nanoencapsulation into liposomes. J. Incl. Phenom. Macrocycl. Chem..

[12] Francolini I., Taresco V., Crisante F., Martinelli A., D’Ilario L., Piozzi A. (2013). Water soluble usnic acid-polyacrylamide complexes with enhanced antimicrobial activity against *Staphylococcus epidermidis*. Int. J. Mol. Sci..

[13] Santos N.P.S., Nascimento S.C., Wanderley M.S., Pontes-Filho N.T., Silva J.F., Castro C.M., Pereira E.C., Silva N.H., Honda N.K., Santos-Magalhães N.S. (2006). Nanoencapsulation of usnic acid: An attempt to improve antitumour activity and reduce hepatotoxicity. Eur. J. Pharm. Biopharm..

[14] Ribeiro-Costa R.M., Alves A.J., Santos N.P., Nascimento S.C., Gonçalves E.C., Silva N.H., Honda N.K., Santos-Magalhães N.S. (2004). In vitro and in vivo properties of usnic acid encapsulated into PLGA-microspheres. J. Microencapsul..

[15] Francolini I., Norris P., Piozzi A., Donelli G., Stoodley P. (2004). Usnic acid, a natural antimicrobial agent able to inhibit bacterial biofilm formation on polymer surfaces. Antimicrob. Agents Chemother..

[16] Labib M.E., Brumlik C.J., Stoodley P., Dukhin S.S., Davidson T., Tabani Y. (2010). The long-term release of antibiotics from monolithic nonporous polymer implants for use as tympanostomy tubes. Colloids Surf A Physicochem Eng. Asp..

[17] Siqueira-Moura M.P., Lira M.C.B., Santos-Magalhães N.S. (2008). Validação de método analítico espectrofotométrico UV para determinação de ácido úsnico em lipossomas. Rev. Bras. Cienc. Farm..

[18] Göke K., Lorenz T., Repanas A., Schneider F., Steiner D., Baumann K., Bunjes H., Dietzel A., Finke J.H., Glasmacher B. (2018). Novel strategies for the formulation and processing of poorly watersoluble drugs. Eur. J. Pharm. Biopharm..

[19] Poecheim J., Graeser K.A., Hoernschemeyer J., Becker G., Storch K., Printz M. (2018). Development of stable liquid formulations for oligonucleotides. Eur. J. Pharm. Biopharm..

[20] Zanolla D., Perissutti B., Passerini N., Chierotti M.R., Hasa D., Voinovich D., Gigli L., Demitri N., Geremia S., Keiser J. (2018). A new soluble and bioactive polymorph of praziquantel. Eur. J. Pharm. Biopharm..

[21] Yang Y., Bae W.K., Lee J.Y., Choi Y.J., Lee K.H., Park M.S., Yu Y.H., Park S.Y., Zhou R., Taş İ. (2018). Potassium usnate, a water-soluble usnic acid salt, shows enhanced bioavailability and inhibits invasion and metastasis in colorectal cancer. Sci. Rep..

[22] Martins M.C.B., Silva M.C., Silva L.R.S., Lima V.L.M., Pereira E.C., Falcão E.P., Melo A.M.M.A., Silva N.H. (2014). Usnic acid potassium salt: An alternative for the control of *Biomphalaria glabrata* (Say, 1818). PLoS ONE.

[23] Araújo H.D.A., Melo A.M.M.A., Siqueira W.N., Martins M.C.B., Aires A.L., Albuquerque M.C.P.A., Silva N.H., Lima V.L.M. (2018). Potassium usnate toxicity against embryonic stages of the snail *Biomphalaria glabrata* and *Schistosoma mansoni* Cercariae. Acta Trop..

[24] Araújo H.D.A., Silva L.R.S., Siqueira W.N., Fonseca C.S.M., Silva N.H., Melo A.M.M.A., Martins M.C.B., Lima V.L.M. (2018). Toxicity of usnic acid from *Cladonia substellata* (Lichen) to embryos and adults of *Biomphalaria glabrata*. Acta Trop..

[25] Araújo H.D.A., Aires A.L., Soares C.L.R., Brito T.G.S., Nascimento W.M., Martins M.C.B., Silva T.G., Brayner F.A., Alves L.C., Silva N.H. (2018). Usnic acid potassium salt from *Cladonia substellata* (Lichen): Synthesis, cytotoxicity and in vitro anthelmintic activity and ultrastructural analysis against adult worms of *Schistosoma mansoni*. Acta Trop..

[26] Huneck S., Yoshimura I. (1996). Identification of Lichen Substances.

[27] Ribár B., Kapor A., Argay G., Engel P., Djarmati Z., Jankov R.M. (1993). Crystal structure of usnic acid sodium salt 2 1/2 hydrate. J. Cryst. Spectr. Res..

[28] Frisch M.J., Trucks G.W., Schlegel H.B., Scuseria G.E., Robb M.A., Cheeseman J.R., Montgomery J.A., Vreven T., Kudin K.N., Burant J.C. (2009). Gaussian 03, Revision c.02.

[29] Buemi G., Zuccarello F. (1990). Molecular conformations, hydrogen-bond strengths and electronic structure of usnic acid: An AM1 and CNDO/S study. J. Mol. Struc. Theochem..

[30] Galasso V. (2010). Probing the molecular and electronic structure of the lichen metabolite usnic acid: A DFT study. Chem. Phys..

[31] Bouasla A., Bouasla I. (2017). Ethnobotanical survey of medicinal plants in northeastern of Algeria. Phytomedicine.

[32] Cechinel-Filho V., Yunes R.A. (1998). Estratégias para a obtenção de compostos farmacologicamente ativos a partir de plantas medicinais: Conceitos sobre modificação estrutural para otimização da atividade. Quím. Nova..

[33] Ahmad M., Lim C.P., Akowuah G.A., Ismail N.N., Hashim M.A., Hor S.Y., Ang L.F., Yam M.F. (2013). Safety assessment of standardised methanol extract of *Cinnamomum burmannii*. Phytomedicine.

[34] Nascimento D.K., Souza I.A., Oliveira A.F., Barbosa M.O., Santana M.A., Pereira Júnior D.F., Lira E.C., Vieira J.R. (2016). Phytochemical screening and acute toxicity of aqueous extract of leaves of *Conocarpus erectus* Linnaeus in swiss albino mice. An. Acad. Bras. Cienc..

[35] Sigma-Aldrich, Safety Data Sheet. https://www.sigmaaldrich.com/MSDS/MSDS/DisplayMSDSPage.do?country=BR&language=pt&productNumber=329967&brand=ALDRICH&PageToGoToURL=https%3A%2F%2Fwww.sigmaaldrich.com%2Fcatalog%2Fsearch%3Fterm%3D7562-610%26interface%3DCAS%2520No.%26N%3D0%26mode%3Dpartialmax%26lang%3Dpt%26region%3DBR%26focus%3Dproduct.

[36] OECD—Organisation for Economic Co-operation and Development (2001). Guidelines for the Testing of Chemicals, OECD 423. Acute Oral Toxicity-Acute Toxic Class Method.

[37] Han D., Matsumaru K., Rettori D., Kaplowitz N. (2004). Usnic acid-induced necrosis of cultured mouse hepatocytes: Inhibition of mitochondrial function and oxidative stress. Biochem. Pharmacol..

[38] Pramyothin P., Janthasoot W., Pongnimitprasert N., Phrukudom S., Ruangrungsi N. (2004). Hepatotoxic effect of (+)usnic acid from *Usnea siamensis* Wainio in rats, isolated rat hepatocytes and isolated rat liver mitochondria. J. Ethnopharmacol..

[39] Joseph A., Lee T., Moland C.L., Branham W.S., Fuscoe J.C., Leakey J.E.A., Allaben W.T., Lewis S.M., Ali A.A., Desai V.G. (2009). Effect of (+)-usnic acid on mitochondrial functions as measured by mitochondria-specific oligonucleotide microarray in liver of B6C3F1 mice. Mitochondrion.

[40] Neff G.W., Reddy K.R., Durazo F.A., Meyer D., Marrero R., Kaplowitz N. (2004). Severe hepatotoxicity associated with the use of weight loss diet supplements containing ma huang or usnic acid. J. Hepatol..

[41] Sanchez W., Maple J.T., Burgart L.J., Kamath P.S. (2006). Severe hepatotoxicity associated with use of a dietary supplement containing usnic acid. Mayo Clin. Proc..

[42] Moura M.R.L., Reyes F.G. (2002). Interação fármaco-nutriente: Uma revisão. Rev. Nutr..

[43] Radulovic L.L., Cilla D.D., Posvar E.L., Sedman A.J., Whitfield L.R. (1995). Effect of food on the bioavailability of atorvastatin, an HMG-CoA reductase inhibitor. J. Clin. Pharmacol..

[44] Lavelle J., Follansbee S., Trapnell C.B., Buhles W.C., Griffy K.G., Jung D., Dorr A., Connor J. (1996). Effect of food on the relative bioavailability of oral ganciclovir. J. Clin. Pharmacol..

[45] Oliveira A.M., Nascimento M.F., Ferreira M.R.A., Moura D.F., Souza T.G.S., Silva G.C., Ramos E.H.S., Paiva P.M.G., Medeiros P.L., Silva T.G. (2016). Evaluation of acute toxicity, genotoxicity and inhibitory effect on acute inflammation of an ethanol extract of *Morus alba* L. (Moraceae) in mice. J. Ethnopharmacol..

[46] Oliveira A.M., Mesquista M.S., Silva G.C., Silva E.O., Medeiros P.L., Paiva P.M.G., Souza I.A., Napoleão T.H. (2015). Evaluation of toxicity and antimicrobial activity of an ethanolic extract from leaves of *Morus alba* L. (Moraceae). Evid. Based Complement. Alternat. Med..

[47] Oliveira A.M., Freire M.O.L., Silva W.A.V., Ferreira M.R.A., Paiva P.M.G., Soares L.A.L., Medeiros P.L., Carvalho B.M., Napoleão T.H. (2018). Saline extract of *Pilosocereus gounellei* stem has antinociceptive effect in mice without showing acute toxicity and altering motor coordination. Regul. Toxicol. Pharmacol..

[48] Kumar V., Abbas A.K., Fausto N., Aster J.C. (2010). Robbins E Cotran, Bases Patológicas Das Doenças.

[49] Le Bars D., Gozariu M., Cadden S.W. (2001). Animal models of nociception. Pharmacol. Rev..

[50] Rocha A.P., Kraychete D.C., Lemonica L., Carvalho L.R., Barros G.A., Garcia J.B., Sakata R.K. (2007). Pain: Current aspects on peripheral and central sensitization. Rev. Bras. Anestesiol..

[51] Lewanowitsch T., Miller J.H., Irvine R.J. (2006). Reversal of morphine, methadone and heroin induced effects in mice by naloxone methiodide. Life Sci..

[52] Bershad A.K., Miller M.A., Norman G.J., Wit H. (2018). Effects of opioid- and non-opioid analgesics on responses to psychosocial stress in humans. Horm. Behav..

[53] McNamara C.R., Mandel-Brehm J., Bautista D.M., Siemens J., Deranian K.L., Zhao M., Hayward N.J., Chong J.A., Julius D., Moran M.M. (2007). TRPA1 mediates formalin-induced pain. Proc. Natl. Acad. Sci. USA.

[54] Zhai C., Liu Q., Zhang Y., Wang S., Zhang Y., Li S., Qiao Y. (2014). Identification of natural compound carnosol as a novel TRPA1 receptor agonist. Molecules.

[55] Wang S., Zhai C., Zhang Y., Yu Y., Zhang Y., Ma L., Li S., Qiao Y. (2016). Cardamonin, a novel antagonist of hTRPA1 cation channel, reveals therapeutic mechanism of pathological pain. Molecules.

[56] Marenich A.V., Cramer C.J., Truhlar D.G. (2009). Universal solvation model based on solute electron density and on a continuum model of the solvent defined by the bulk dielectric constant and atomic surface tensions. J. Phys. Chem. B.

[57] Breneman C.M., Wiberg K.B. (1990). Determining atom-centered monopoles from molecular electrostatic potentials. The need for high sampling density in formamide conformational analysis. J. Comput. Chem..

[58] Malone M.H., Wagner H., Wolf P. (1977). Pharmacological Approaches to Natural Product, Screening and Evaluation. New Natural Products and Plant Drugs with Pharmacological Biological or Therapeutical Activity.

[59] Kamarudin N., Hisamuddin N., Ong H.M., Azmi A.F.A., Leong S.W., Abas F., Sulaiman M.R., Mossadeq W.M.S. (2018). Analgesic effect of 5-(3,4-Dihydroxyphenyl)-3-hydroxy-1-(2-hydroxyphenyl)penta-2,4-dien-1-one in experimental animal models of nociception. Molecules.

[60] Ping C.P., Mohamad T.A.S.T., Akhtar M.N., Perimal E.K., Akira A., Israf Ali D.A.I., Sulaiman M.R. (2018). Antinociceptive effects of cardamonin in mice: Possible involvement of TRPV_1_, glutamate, and opioid receptors. Molecules.

[61] Hunskaar S., Hole K. (1987). The formalin test in mice: Dissociation between inflammatory and non-inflammatory pain. Pain.

