# Supplementary material for: Usnic Acid Potassium Salt: Evaluation of the Acute Toxicity and Antinociceptive Effect in Murine Model
Source: Molecules. 2019 May 28;24(11):2042. doi: 10.3390/molecules24112042 (PMC6600509; doi:10.3390/molecules24112042)
Supplement: Supplementary file 1 [file molecules-24-02042-s001.pdf]

Supplementary Files

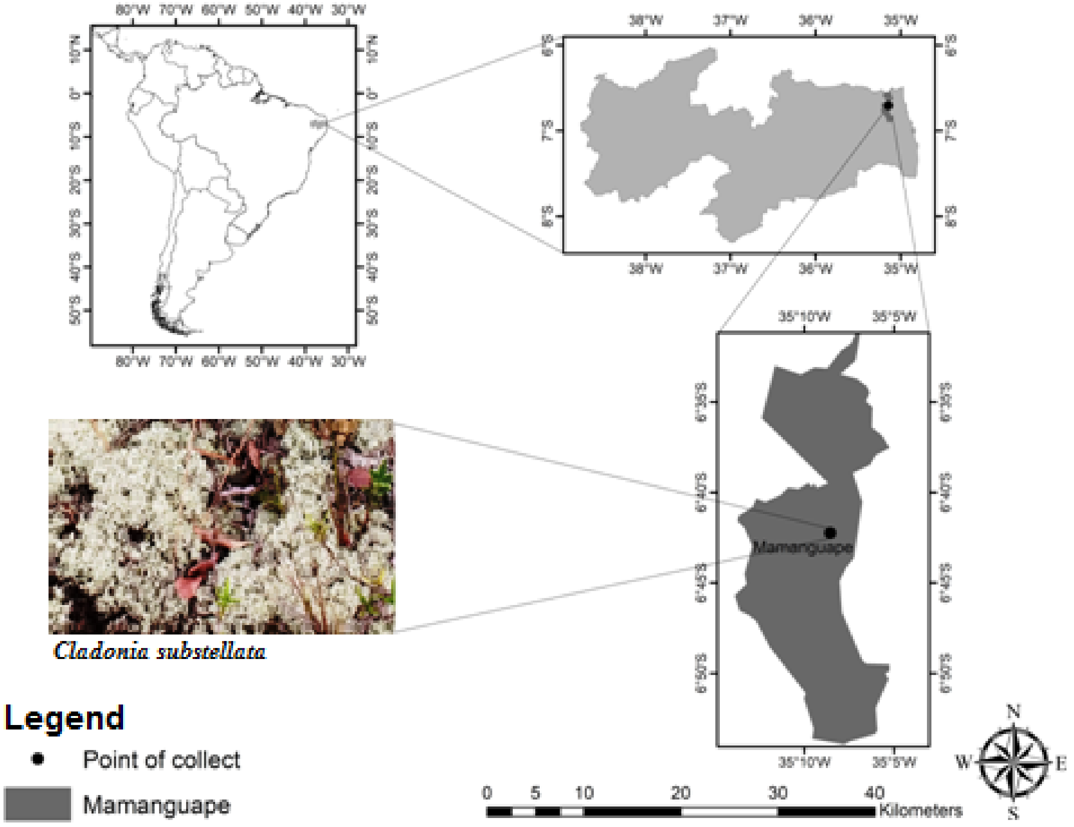

Figure 1. Map of Mamanguape (State of Paraíba - Brazil), showing the occurrence area of lichens.

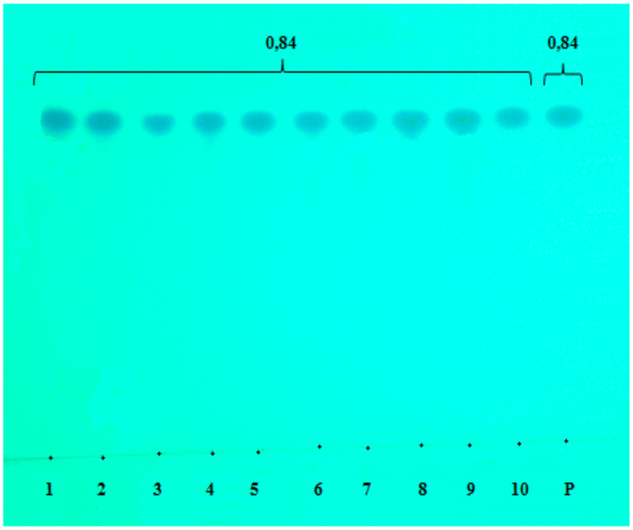

Figure 2. Thin Layer Chromatography (TLC) indicating the presence of a single band under ultraviolet light (254 nm) of the purified samples (points 1-10) compared to the standard sample (P) of Merck's usnic acid.

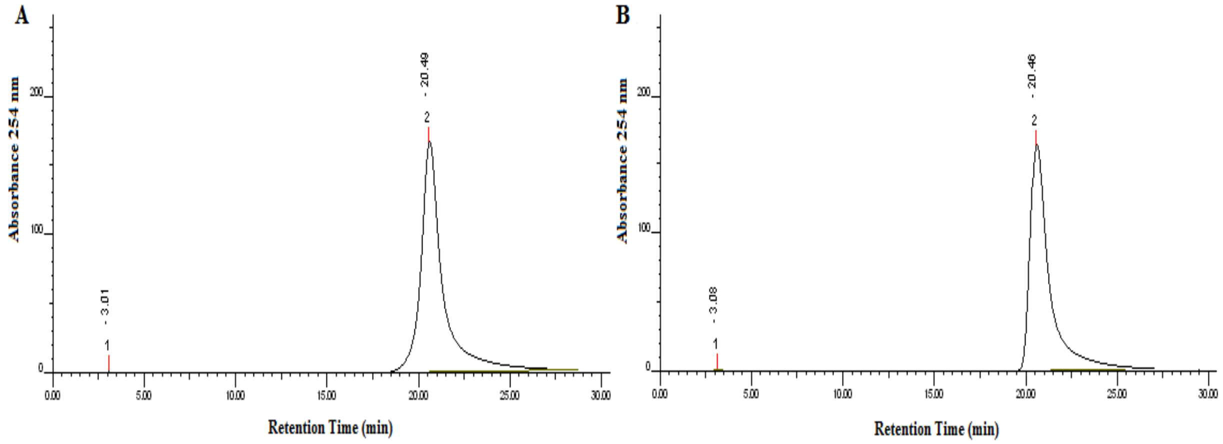

Figure 3. High Performance Liquid Chromatogram (HPLC). A, Standard of Merk RT-20.49 (A) - (99.8% purity) and purified usnic acid from *C. substellata* RT-20.46 (B) - (99.6% purity).

Chemical Analysis of Usnic Acid (UA) and Usnic Acid Potassium Salt (PS-UA)

Confirmation of the structure of UA was performed by proton nuclear magnetic resonance (^{1}H-NMR), Carbon (^{13}C-NMR) and Infrared (IR) spectra. The data are described as follows: ^{1}H-NMR: (400 MHz, acetone-d6) δ_H (H; *mult.;* int.): 1.76 (3H; s, CH_3-13), 2.15 (3H; s, CH_3-16), 2.66 (3H; s, CH_3-15), 2.68 (3H; s, CH_3-18), 5.98 (1H; s, C-4-H), 11.02 (1H; s, C-10- OH), 13.31 (1H; s, C-8-OH), and 18.85 (1H; s, C-3-OH); ^{13}C-NMR: (400 MHz, acetone-d6) δ_C (C; *mult.;* int.): C-1: 198.05, C-2: 191.70, C-3: 157.50, C-4: 98.32, C-5: 101.53, C-6: 76.99, C-7: 155.20, C-8: 163.89, C-9: 103.94, C-10: 179.38, C-11: 109.34, C-12: 59.06, C-13: 27.87, C-14: 200.30, C-15: 32.10, C-16: 7.52, C-17: 201.76 and C-18: 31.25; and IR (KBr): 3090 (ν C-H Ar), 3005 (ν_{as} CH_3), 2925 (ν CH_3), 1695 (ν C=0), 1635, 1550 (ν C=C Ar), 1446 (δ_{as} CH_3) and 1385 (δ_s CH_3) cm^{-1}.

Confirmation of the structure of PS-UA was performed by ^{1}H-NMR (Figure 4), IR (Figure 5) and C H elemental analysis, from which the following data were obtained.

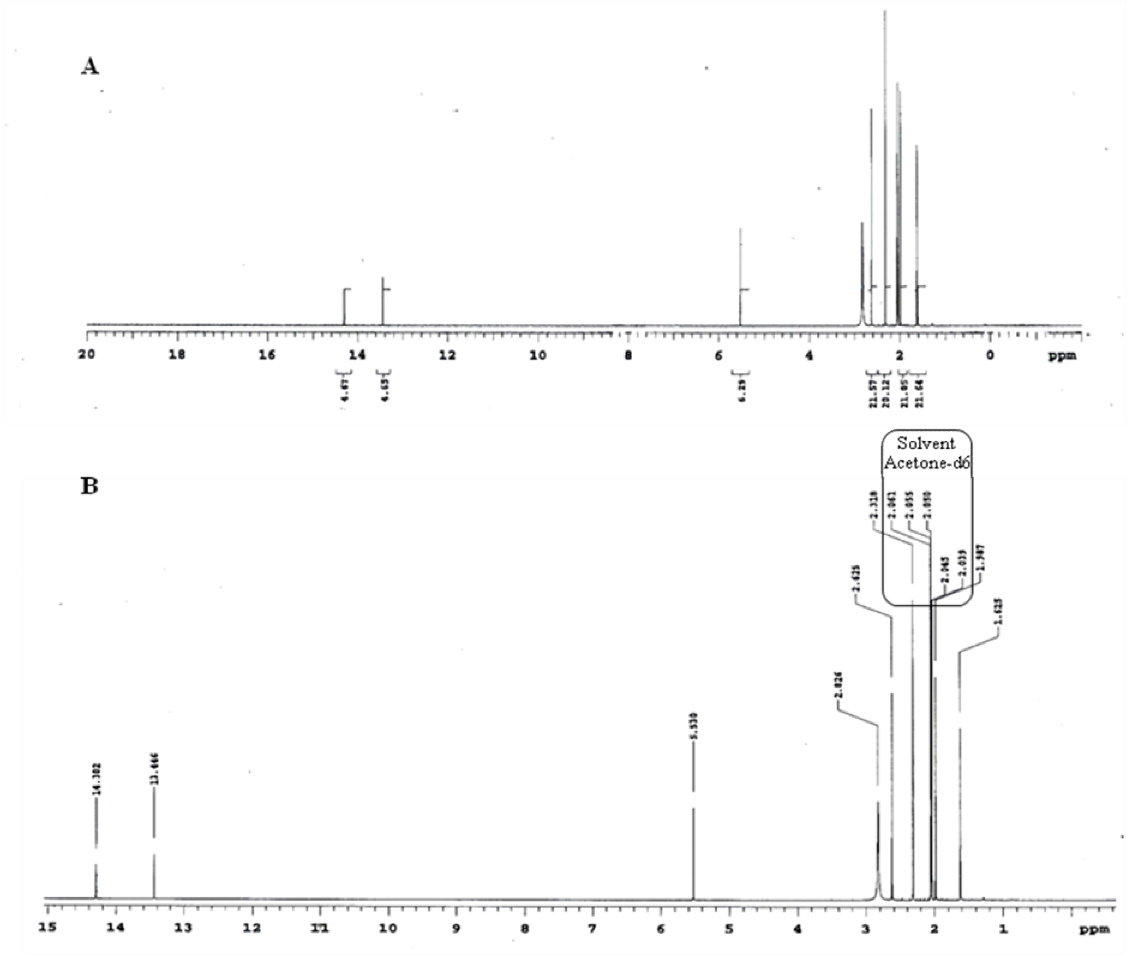

Figure 4. Spectroscopy of purified PS-UA. (A) integration of protons peaks area. (B) Chemical displacement of protons. The data are described as follows: ^{1}H-NMR: (400 MH_z, acetone- d_6) δ_H: 1.62 (3H, s, CH_3-13), 1.98 (3H; s, CH_3-16), 2.62 (3H; s, CH_3-15), 2.82 (3H; s, CH_3-18); 5.53 (1H; s, C-4-H), 13.44 (1H; s, C-10-OH), 14.30 (1H; s, C-3-OH).

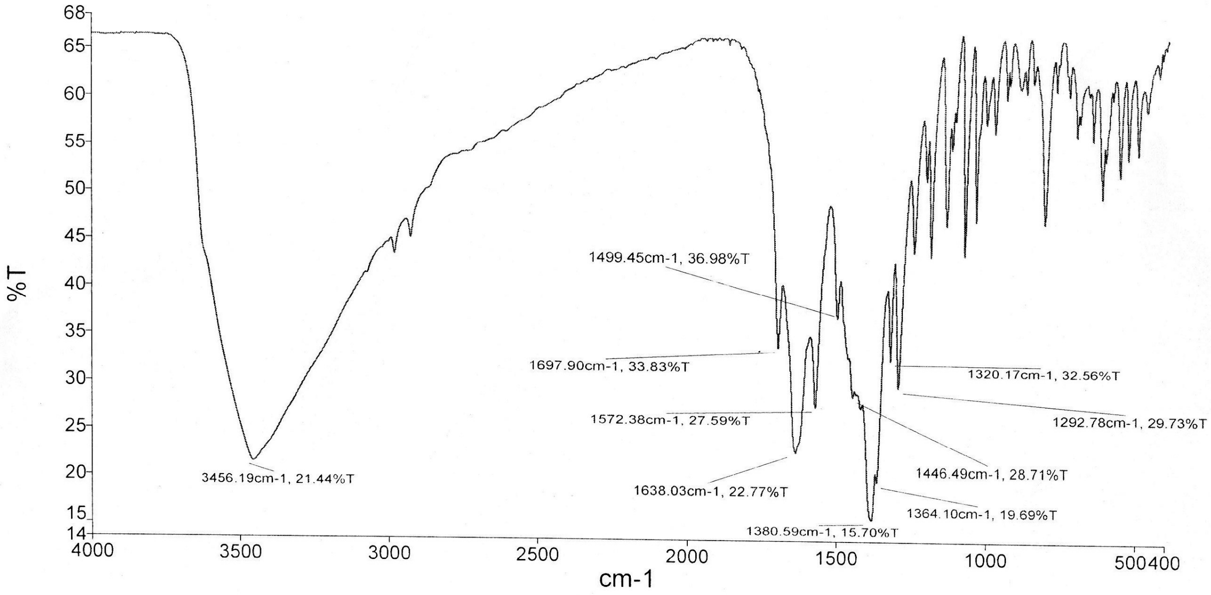

Figure 5. Spectroscopy of purified PS-UA. The spectrum corresponds to % of transmittance versus frequency of vibration. The data are described as follows: IR (KBr): 3456 (ν C-OH), 3096 (ν C-H Ar), 2989 (ν_{as} CH_3), 2929 (ν_s CH_3), 1697 (ν C=0), 1638; 1572 (ν C=C Ar), 1446 (δ_{as} CH_3), 1380 (δ_s CH_3), 1150 and approx. 1070 (ν C-0-C) cm^{-1}.

Elemental analysis: Found: C 51.49%; H 4,69%; N 0.08%; calculated for $C_{18}H_{16}KO_7^-.2H_2O$: C 51.54%; H 4.81%.
